# Occipital Alpha Activity during Stimulus Processing Gates the Information Flow to Object-Selective Cortex

**DOI:** 10.1371/journal.pbio.1001965

**Published:** 2014-10-21

**Authors:** Johanna M. Zumer, René Scheeringa, Jan-Mathijs Schoffelen, David G. Norris, Ole Jensen

**Affiliations:** 1 Radboud University Nijmegen, Donders Institute for Brain, Cognition and Behaviour, Centre for Cognitive Neuroimaging, Nijmegen, The Netherlands; 2 Max Planck Institute for Psycholinguistics, Nijmegen, The Netherlands; University of Oregon, United States of America

## Abstract

Given the limited processing capabilities of the sensory system, it is essential that attended information is gated to downstream areas, whereas unattended information is blocked. While it has been proposed that alpha band (8–13 Hz) activity serves to route information to downstream regions by inhibiting neuronal processing in task-irrelevant regions, this hypothesis remains untested. Here we investigate how neuronal oscillations detected by electroencephalography in visual areas during working memory encoding serve to gate information reflected in the simultaneously recorded blood-oxygenation-level-dependent (BOLD) signals recorded by functional magnetic resonance imaging in downstream ventral regions. We used a paradigm in which 16 participants were presented with faces and landscapes in the right and left hemifields; one hemifield was attended and the other unattended. We observed that decreased alpha power contralateral to the attended object predicted the BOLD signal representing the attended object in ventral object-selective regions. Furthermore, increased alpha power ipsilateral to the attended object predicted a decrease in the BOLD signal representing the unattended object. We also found that the BOLD signal in the dorsal attention network inversely correlated with visual alpha power. This is the first demonstration, to our knowledge, that oscillations in the alpha band are implicated in the gating of information from the visual cortex to the ventral stream, as reflected in the representationally specific BOLD signal. This link of sensory alpha to downstream activity provides a neurophysiological substrate for the mechanism of selective attention during stimulus processing, which not only boosts the attended information but also suppresses distraction. Although previous studies have shown a relation between the BOLD signal from the dorsal attention network and the alpha band at rest, we demonstrate such a relation during a visuospatial task, indicating that the dorsal attention network exercises top-down control of visual alpha activity.

## Abstract 2

A simultaneous EEG-fMRI study demonstrates that alpha-band activity in early visual cortex is associated with gating visual information to downstream regions, boosting attended information and suppressing distraction.

## Author Summary

In complex environments, our sensory systems are bombarded with information. Only a fraction of this information is processed, whereas most is ignored. As such, our brain must rely on powerful mechanisms to filter the relevant information. It has been proposed that alpha band oscillations (8–13 Hz) gate task-relevant visual information to downstream areas and supress irrelevant visual information. We tested this hypothesis in a study that combined electroencephalography (EEG) and functional MRI (fMRI) recordings. From the EEG, we directly measured alpha band oscillations in early visual regions. Using fMRI, we quantified neuronal activity in downstream regions. The participants performed a spatial working memory task that required them to encode pictures of objects presented in the left field of view while ignoring objects in the right field (or vice versa). We found that suppression of alpha band activity in visual areas opened the gate for relevant visual information to be routed to downstream regions. Conversely, an increase in alpha oscillations suppressed visual information that was irrelevant to the task. These findings suggest that alpha band oscillations are directly involved in boosting attended information and suppressing distraction in the ventral visual stream.

## Introduction

Our sensory system receives much more information than can be possibly processed. Selective attention serves to overcome this problem by gating attended information and, importantly, by blocking unattended information [1]. Oscillatory brain activity has been proposed to control the communication between brain regions [2]–[5]. Oscillatory power increases (or decreases) are presumably due to synchronization (or desynchronization) of many neurons in a local patch of cortex [6]. Activity in the alpha band, which has been shown to be related to inhibition of distraction, is strongly present even in the absence of stimuli and is predictive of behavior in both humans [7]–[17] and monkeys [18]–[21]. Further, an increase in alpha band activity is correlated with a decrease in both neuronal excitability, gamma band activity, and the blood-oxygenation-level-dependent (BOLD) signal [22]–[28]. Consequently, it has been proposed that alpha band activity serves to route information to downstream regions by inhibiting neuronal processing in task-irrelevant regions [11],[29],[30]. This allows task-relevant regions to communicate. Although the trial-by-trial effect of anticipatory alpha modulation on *behavior* has been demonstrated [7],[8], the direct effect of alpha modulation during stimulus processing on neuronal activity in downstream regions remains unexplored. We therefore tested this hypothesis by correlating single trial activity recorded simultaneously by electroencephalography (EEG) and functional magnetic resonance imaging (fMRI). Specifically, we investigated whether oscillatory neural oscillations in visual regions recorded by EEG during target evaluation and object encoding predicted representationally specific activation and deactivation in downstream regions as detected by fMRI.

While the posterior alpha activity presumably is under top-down control, it remains unclear which brain regions or networks exercise the control. Combined EEG and transcranial magnetic stimulation studies have implicated the intraparietal sulcus (IPS) and frontal eye field (FEF) in the top-down control [31]. This is consistent with combined EEG/fMRI studies demonstrating that resting state alpha power correlated negatively with the dorsal attention network, including both FEF and IPS [26],[32],[33]. Other resting state fMRI/EEG studies have demonstrated a positive correlation between alpha activity and the precuneus and other regions of the default mode network [34],[35]. We also set out to identify which networks are associated with the control of the occipital alpha activity in a spatial attention task by correlating the task-modulated occipital alpha power with the BOLD signal. In particular, we hypothesized that the dorsal attention network can act to engage occipital cortex by actively suppressing the visual alpha activity.

**Figure 1:**
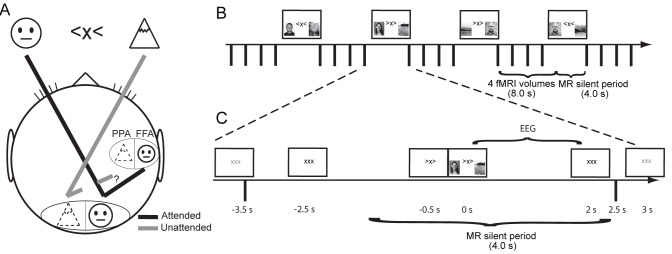
Task design and hypothesis. (A) Participants fixated on a central cross while covertly attending to the left or the right object as indicated by the cue. We predicted enhanced (black lines) processing in the hemisphere contralateral to the attended object in early visual regions and suppressed (gray lines) processing in early visual regions ipsilateral to the attended object. Furthermore, the allocation of spatial attention was expected to result in an enhanced response in object-selective cortex (including FFA and PPA) of the attended object. In this study we asked whether alpha activity measured by EEG in early visual regions gates the object-selective activation as measured by the BOLD signal. (B) Illustration of the one-back visuospatial working memory paradigm involving two object categories (faces and landscapes). Participants were asked to press a button if the cued object matched the object attended in the previous trial. To obtain an EEG signal free from gradient artifacts during the presence of stimuli (4.0 s duration), the fMRI was collected only during the working memory delay period (8.0 s duration). (C) A depiction of an example trial indicates the time range from which alpha power was extracted (EEG), the silent MRI period, and the relative timings between events within a trial. Faces and landscapes depicted here are for illustration and were not used in the experiments.

Our study is based on the finding that when attention is allocated to the left hemifield, the alpha band power decreases in right visual regions, complemented by a relative power increase in left visual regions [8],[9]. This hemispheric alpha power lateralization is reversed when attention is allocated to the right hemifield. A second component in our study is the finding that attention to a face produces an increase of the BOLD contrast in face-selective regions (e.g., fusiform face area [FFA]) [36],[37]. The reverse holds when a landscape is attended, which results in a BOLD signal increase in place-selective regions (e.g., parahippocampal place area [PPA]) [37],[38]. We designed a task in which spatial and object attention were dissociated (Figure 1A). This allowed us to ask whether the modulation of oscillatory brain activity by spatial attention recorded by EEG correlated with the representationally specific BOLD signal modulated by the attended objects.

## Results

### The Paradigm

We developed a two-by-two factorial paradigm in which pictures of faces and landscapes were presented simultaneously in the left and the right hemifields and attention was directed to one hemifield. In the example shown (Figure 1A), participants were required to attend to the cued image (left hemifield), which in this case was a face, while ignoring the landscape presented to the right. Participants had to indicate by a button press whether the cued object matched the object attended in the previous trial; note that the hemifields could be different for matches (as in the last panel of Figure 1B). No responses were required for non-matches.

We recorded simultaneously EEG, eye tracker, and fMRI data in 16 healthy human participants. In each trial, a central cue presented for 0.5 s indicated the direction of attention. This brief cue-target interval allowed some time for alpha lateralization to accrue in preparation for selective spatial attention once the images appeared. Then the object pair was presented for 2 s while the central cue remained (Figure 1C). This long stimulus duration allowed us to probe the alpha activity during stimulus processing, rather than either during the anticipatory cue-target interval or during the one-back working memory delay period. Of 200 trials total, 160 were non-matches, 28 were matches (response required), and the remaining 12 were catch trials. Catch trials were those in which an image was repeated in a subsequent trial but was shown in the unattended hemifield either on the first or second presentation; no response was required. Participants performed the working memory task well (89% hits, 1% false alarms on non-match trials, and 3% false alarms on catch trials).

### Hemispheric Alpha Lateralization Modulated with Spatial Attention in Occipital Cortex

We first characterized the modulation of oscillatory activity in the EEG with spatial attention. Time–frequency representations of power were calculated for the posterior electrodes for individual trials. From these time–frequency representations, we selected the optimal time window across all participants and center frequency for each participant during stimulus presentation in which the modulation of alpha with left versus right attention was greatest. We used this time–frequency window to compute an adaptive spatial inverse filter to reconstruct the neuronal source power over a discretized 3-D grid. At every grid point we computed the alpha modulation index (AMI)
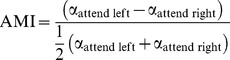
(1)over trials. The individual volumes of power were then normalized to a standard brain prior to averaging.

**Figure 2:**
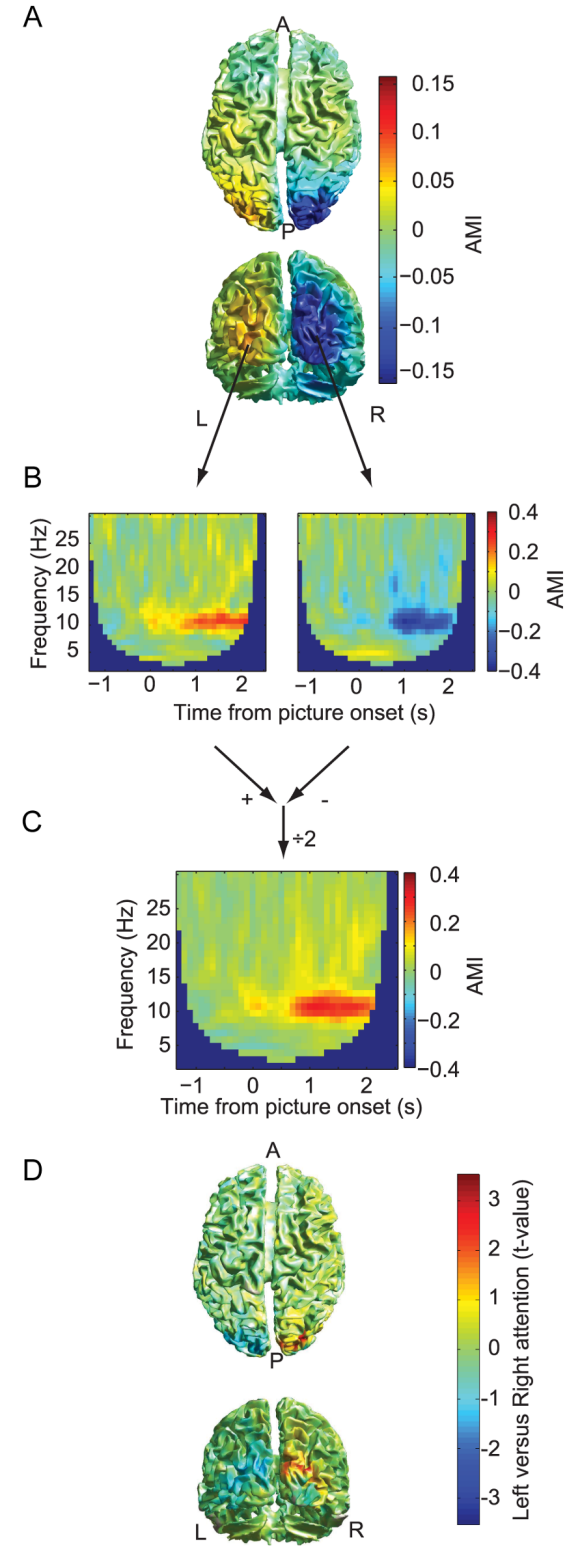
The modulation of visual oscillatory and hemodynamic brain activity with the direction of covert attention. (A) The grand average of the source plot of the alpha band modulation in the early retention interval (0.1–1.9 s after picture stimulus onset) quantified by the AMI (attention left minus attention right). The alpha activity was suppressed in early visual cortex contralateral to the direction of attention, whereas ipsilateral it relatively increased. (B) Time–frequency representations of oscillatory power derived for the sources in left (left panel) and right (right panel) visual cortex. (C) The time–frequency representations combined (left minus right, divided by two) over the two hemispheres demonstrate that the modulation was constrained to the alpha band and was sustained. (D) The grand average of the statistical contrast (*t*-statistic) of hemodynamic activity of attend-left versus attend-right trials. The grand averages of this contrast specific to select sub-regions are shown in Figure S1. A, anterior; L, left; P, posterior; R, right.

Figure 2A shows the AMI (frequency interval including 7–13 Hz, and time intervals including 0.1–1.9 s after stimulus onset) projected onto the surface of a standard brain. This analysis revealed a strong depression in alpha power in the hemisphere contralateral to the cued hemifield and a relative alpha power increase in the ipsilateral hemisphere. The modulation was strongest in the left and right occipital cortices (Montreal Neurological Institute [MNI] coordinates [−29 −88 29] mm and [27 −94 24] mm, located, respectively, within the left middle occipital and right superior occipital regions according to the automated anatomical labeling [39]). Figure 2B shows the time–frequency representation of power of the EEG from the occipital regions for right versus left attention. The combined measure (left minus right) revealed a 30% attention modulation specific to the alpha band emerging ∼500 ms after image onset (Figure 2C).

This demonstrated the feasibility of detecting robust modulations in the alpha band with attention in EEG data collected inside the MRI environment, as well as of detecting persistent alpha lateralization during stimulus presentation despite the overall reduction of bilateral alpha power in response to the visual stimuli. Consistent with previous findings [40]–[42], the localization in occipital regions was complemented by significant modulation with spatial attention of the BOLD signal in early visual areas, specifically in bilateral superior, middle, and inferior occipital cortex, as well as in the right cuneus (Figures 2D and S1). We conclude that hemisphere-specific occipital alpha power was reliably modulated by spatial cueing of covert visual attention, even when visual stimuli were present.

### BOLD Modulations in Object-Selective Cortex with Attention

**Figure 3:**
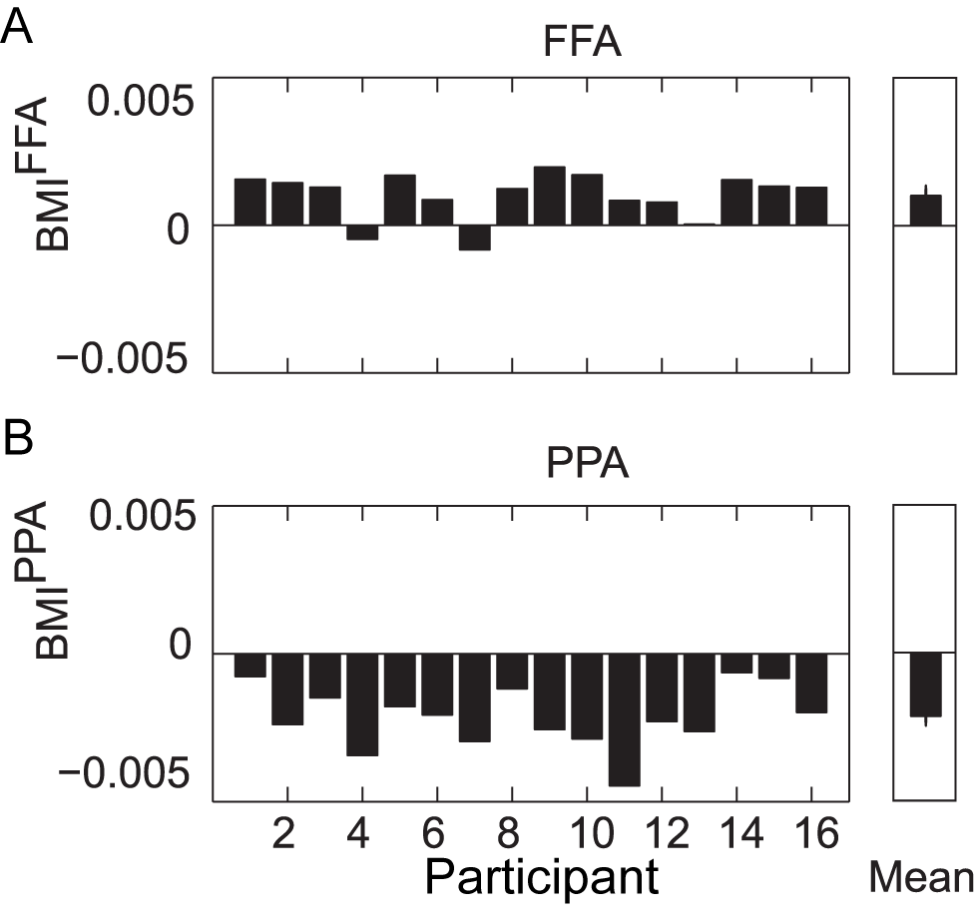
Modulation of BOLD activity with different object types in the attended hemifield. (A) BMI is the relative difference of a given region's activity between two conditions, normalized by the mean of the activity in both conditions (computed as in Equation 2). Error bars are the standard error over participants. Both FFA and PPA showed a BMI significantly different from zero (*p*<0.001). BMI for the FFA is shown per participant (left panel) and as the mean over participants (right panel). (B) BMI for the PPA is shown per participant (left panel) and as the mean over participants (right panel). A visualization of the FFA and PPA regions per participant is shown in Figure S2. The time course of the FFA and PPA regions' BOLD signal is shown in Figure S3.

To characterize the object-selective BOLD activity in each participant, we applied a separate functional localizer task (see Materials and Methods) to identify the regions responding strongest to either faces or landscapes. These regions were mainly located in inferior temporal cortex and included the bilateral FFA and bilateral PPA (Figure S2). We then applied a mask obtained from this localizer of these regions (FFA and PPA) to analyze the BOLD data collected in the attention paradigm (Figure 1B). We defined a BOLD modulation index (BMI) for representational selectivity based on the regions of interest (ROIs)
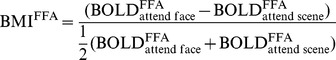
(2)for the FFA and similarly for the PPA. The BMI^FFA^ was larger when a face was attended than when unattended (Figures 3A and S3C), and vice versa for BMI^PPA^ (Figures 3B and S3D), thus reproducing previous findings [43]. Thus, even though attention was cued spatially (rather than categorically [44]), the processing of the object that happened to fall inside this attended hemifield was nevertheless boosted, as indexed by the BOLD response in object-selective cortex.

### Occipital Alpha Power Was Negatively Correlated with the BOLD Signal in Early Visual Cortex

Our analysis so far has demonstrated that the BOLD contrast and alpha power modulations with attention originated from left and right occipital cortices. To relate those measures, we correlated the single trial alpha power during target presentation with the target-evoked BOLD signal from the occipital regions. We observed a negative relationship between alpha power and the occipital BOLD signals for all attention conditions (Figure S4). These results are in line with previous reports [26]–[28] and support the notion that alpha oscillations at the source location serve to inhibit activity.

### Occipital Alpha Power Predicted the Downstream BOLD Signal

**Figure 4:**
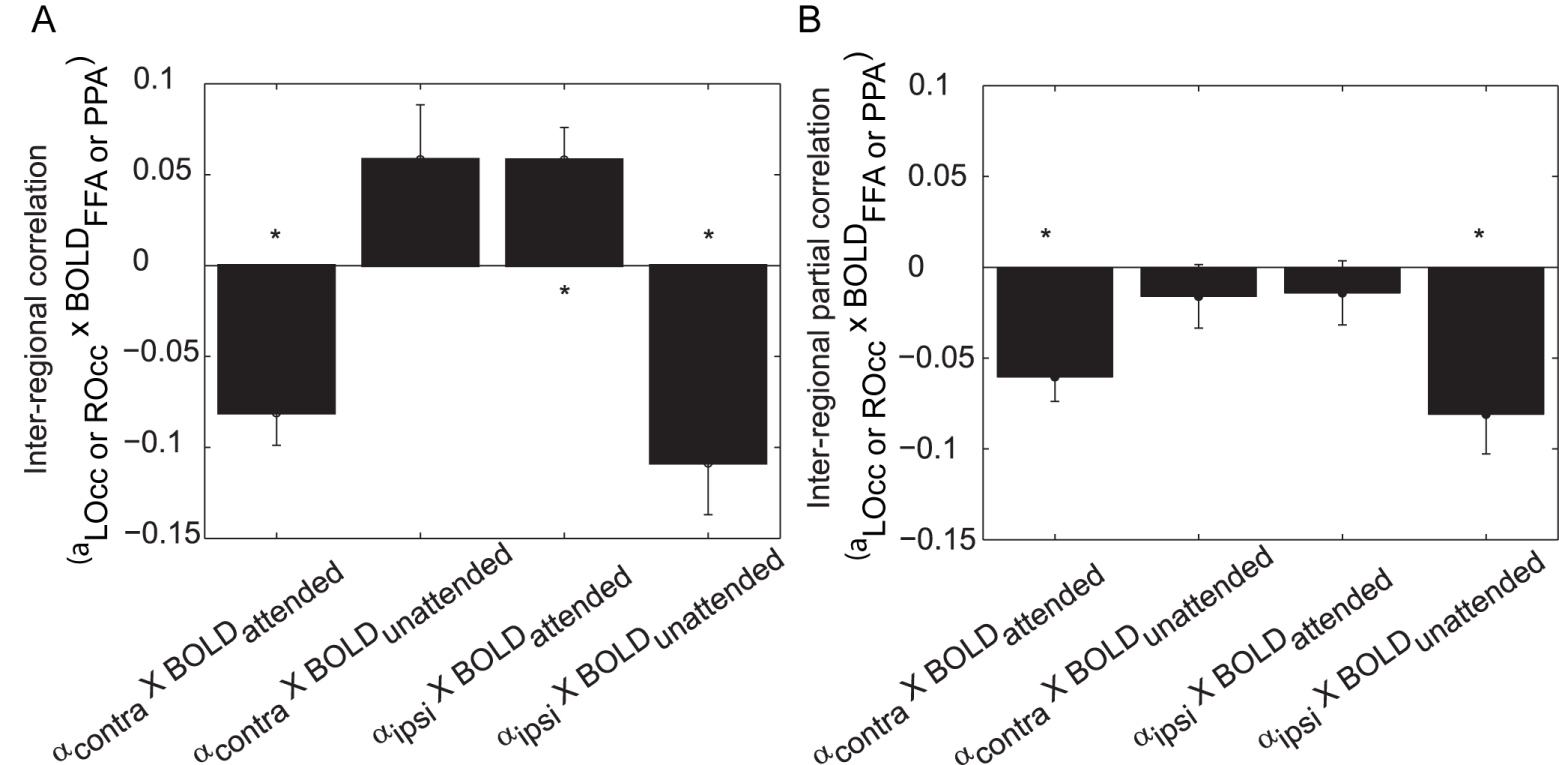
Correlations between hemispheric occipital alpha power and object-selective region BOLD amplitude. (A) Each bar is specific to whether the alpha power is recorded from the hemisphere contra- or ipsilateral to the attended direction, and whether the BOLD amplitude is from the region corresponding to the attended or unattended object type. The Spearman correlation was computed over trials within a participant; error bars indicate standard error over participants. All mean correlations over participants were significantly different from zero (*p<0.05). (B) An additional partial Spearman correlation was computed to account for the alpha power of the other hemisphere and the BOLD response of the other object-selective region (*p<0.05). The first bar depicts a negative correlation, indicating that a decrease in alpha power contralateral to the attended object predicted an increase in the ventral BOLD signal for the attended object. The last bar also depicts a negative correlation, indicating that the alpha power ipsilateral to the attended object predicted a decrease in the ventral BOLD signal for the unattended object. The correlations of occipital alpha power with occipital BOLD activity are shown in Figure S4. LOcc, left occipital; ROcc, right occipital.

Next, we set out to characterize the relationship between task-specific modulations in the alpha band and the representationally specific modulations in the BOLD signal. We did this by correlating over single trials the alpha power—from the same 0.1–1.9-s time window during stimulus processing described above—in one hemisphere (contralateral or ipsilateral to the attended direction) with the stimulus-evoked BOLD activity in an object-selective region (corresponding either to the attended or unattended object). We found that the alpha power in the hemisphere contralateral to the attended direction correlated negatively with the representationally specific BOLD signal in the object-selective cortex of the attended object (Figure 4A, first bar; *p*<0.001). Using Figure 1A as an example where a participant must attend to a face in the left hemifield, this finding indicated that in trials where the right hemisphere alpha power decreased, the BOLD signal in FFA increased. Note that we combined trials for which faces were attended 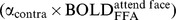
with trials for which landscapes were attended 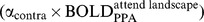. The correlations demonstrated that trials with lower alpha power contralateral to the attended hemifield, which on average was already decreased compared to when ignored stimuli were processed contralaterally, predicted a stronger representationally specific BOLD response to the attended object in the ventral stream. Correspondingly, the contralateral alpha power positively correlated with the BOLD response in the object-selective area coding for the unattended objects (Figure 4A, second bar; *p* = 0.06).

Importantly, we found that the ipsilateral alpha power correlated negatively with the BOLD response for the object-selective area responding to the unattended object (Figure 4A, fourth bar; *p* = 0.001). Continuing with the example from Figure 1A with a landscape ignored in the right hemifield, this finding indicated that in trials where the left hemisphere alpha power increased, the BOLD signal in PPA decreased. We also found that the ipsilateral alpha power correlated positively with the representationally specific BOLD increase for the attended object (Figure 4A, third bar; *p* = 0.004).

It could be that activities from the four measures (contra- and ipsilateral alpha power, and FFA and PPA BOLD signal) were all co-modulated. To investigate this, we computed partial correlations to isolate the hemisphere- and object-region-dependent correlations (see Materials and Methods). After controlling for the modulations in the other-hemisphere alpha power (e.g., controlling for ipsilateral alpha power when considering the contralateral visual regions) and the BOLD response in the other object-specific region (e.g., controlling for the FFA BOLD signal when considering the PPA BOLD signal), only the *negative* correlations remained significant (Figure 4B; first and fourth bar).

The time–frequency results in Figure 2 showed that the strongest modulation was in the alpha band; however, trial-by-trial coupling (and hence gating) may have also occurred between power in another frequency band and the object-selective BOLD response. We computed the same correlations as in Figure 4 using the beta band power and theta band power. Only a negative correlation between contralateral beta power and the BOLD signal of the attended region was observed; however, this did not remain significant when controlling for the other regions' activity. No task-induced effects were found in the gamma band range; thus, the gamma band was not further tested for correlations with BOLD signal.

### The BOLD Signal in the Dorsal Attention Network Correlated with Occipital Alpha Power

**Figure 5:**
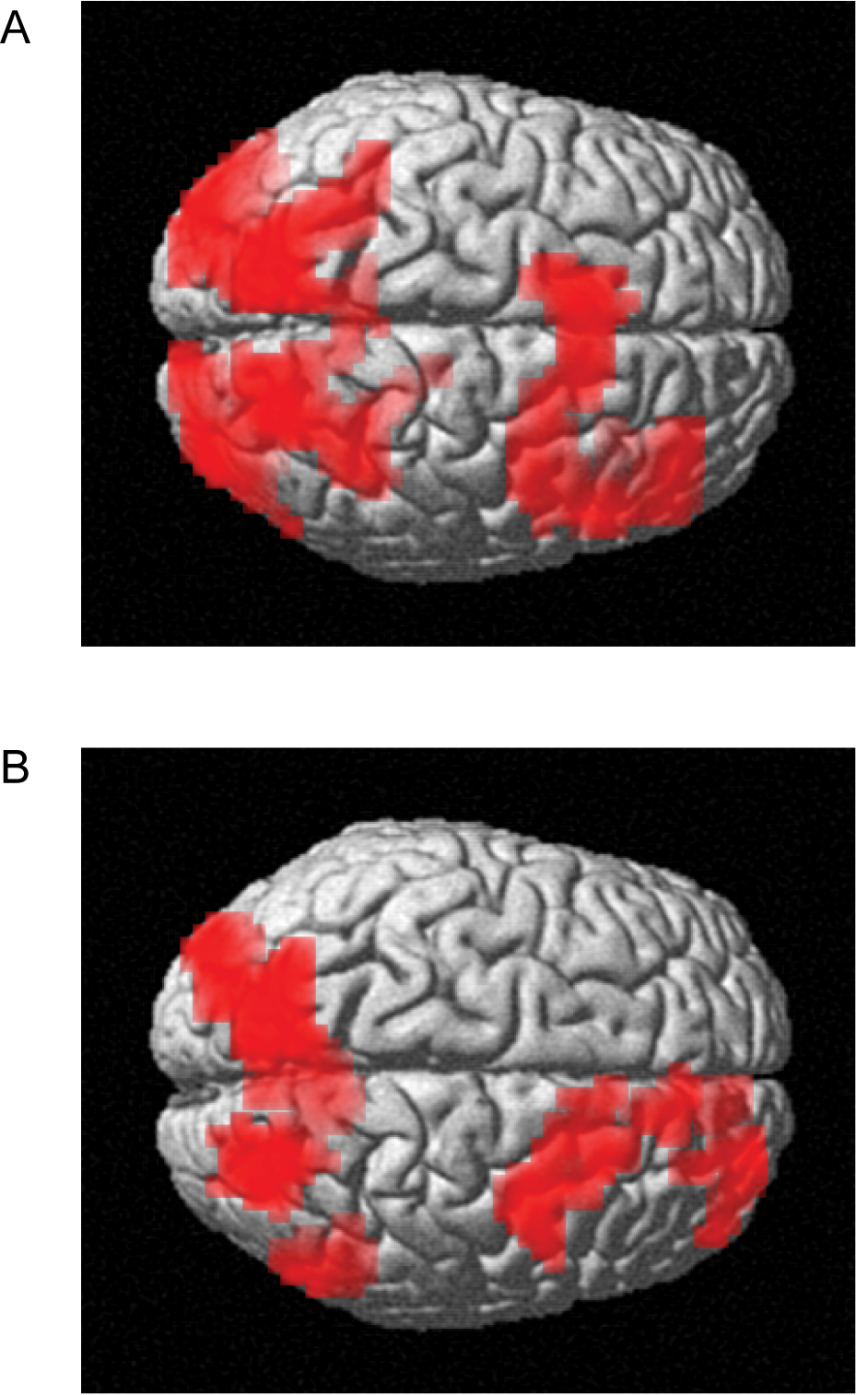
Regions showing significant relationships with occipital alpha power, independent of direction or object of attention. (A) The BOLD activity inversely related to the left and right occipital alpha power for combined left and right attention. The dorsal attention network including the IPS and the right FEF emerges from this analysis (*p*<0.005 uncorrected followed by *p*<0.05 FWE cluster-level corrected). (B) The BOLD activity inversely related to the ipsilateral alpha power only. Again, the dorsal attention network emerges. No significant effects were identified when considering the contralateral alpha power only (*p*<0.005 uncorrected followed by *p*<0.05 FWE cluster-level corrected). The control analysis of BOLD activity assessing which regions were associated with the task of spatial attention is shown in Figure S5.

We then set out to identify nonvisual regions in which the BOLD signal correlated with the occipital alpha activity. The aim was to identify putative regions or a network involved in the top-down control of the posterior alpha activity. We established a general linear model (GLM) with regressors to model the task-related variance, non-task-related variance, and individual trial alpha-power-related variance. See Materials and Methods for details. Using a spatial filter, we extracted the single trial alpha band power from the left and right occipital areas (see Figure 2). Each value per hemisphere was either contra- or ipsilateral to the direction of attention for a given trial. This resulted in four regressors for the alpha activity. When all four alpha regressors were considered together, the bilateral occipital BOLD signals were found to be inversely related (Figure 5A), as expected [25]–[28] and in agreement with the different but related analysis shown in Figure S3. These bilateral occipital BOLD signals were not found to be significantly related to alpha power when ipsilateral only (Figure 5B) or contralateral only (not shown) alpha power was tested.

Interestingly, we also found that the BOLD signal in regions related to the dorsal attention network (right FEF, medial prefrontal cortex, and bilateral IPS) was inversely related to the occipital alpha power, after controlling for task and other non-task variance. This was true when all four alpha power regressors were considered together (Figure 5A), as well as when only the two alpha power regressors corresponding to the ipsilateral hemifield for that trial were considered (Figure 5B) (*p*<0.005 initial threshold followed by *p*<0.05 family-wise error [FWE] cluster-level threshold). No significant relation was found using contralateral alpha power alone. Note that the engagement of prefrontal regions was right dominated. These results suggest that the dorsal attention network modulated the occipital alpha power.

## Discussion

Our findings demonstrate that the visual alpha activity predicted representationally specific activation in downstream regions. We propose that these predictions, indicated by the correlations depicted in Figure 4, reflect a gating mechanism that is achieved by a decrease in the alpha activity in the regions processing spatially attended information and, importantly, by a relative increase in the alpha power in the regions processing unattended information. The modulation of the occipital alpha activity, and especially the alpha activity in the ipsilateral hemisphere processing unattended information, was related to activity in the dorsal attention network.

Previous findings have emphasized that increases in functional connectivity in the *attended* path between occipital and downstream areas serves to allocate attention [45],[46]. This could, for instance, be implemented by a gain increase in the occipital region processing the attended information. Our finding of a relation between the contralateral alpha power and downstream BOLD contrast increase in attended regions is in support of a gain increase in early regions with attention. However, when allocating visual attention, it is just as important to suppress unattended information [14],[15],[43]. We have demonstrated that the suppression of unattended information is reflected by active inhibition via increased alpha band activity in the unattended visual stream (see also Figure 1A, dashed gray line). Our findings add support to the notion that alpha band activity serves an active role in the allocation of resources. We suggest that the mechanism of this gating by alpha activity is via phasic manipulation of higher frequency oscillations in the gamma band (e.g., [24]). In particular, the increase in alpha activity is important for the routing of information by depressing irrelevant processing of sustained stimulation, which is in stark contrast to the view that posterior alpha activity reflects idling or drowsiness. Our task did not have a “baseline” condition, so it is not clear whether the ipsilateral alpha power increased or decreased with respect to a lack of directed attention. Nevertheless, the modulation was robust with respect to spatial attention. Please see Jensen et al. [47] for an extensive discussion of the role of ipsilateral alpha oscillations. In future work it would be of interest to investigate whether the routing by alpha activity is complemented by increased phase synchronization between early visual (contralateral to the attended stimulus) and downstream ventral areas. Indeed, recordings in monkeys have demonstrated phase synchronization in the alpha band between ventral areas during the allocation of attention; interestingly, the synchronization appeared to be coordinated by the pulvinar [18].

Our main finding of correlations between occipital alpha power and downstream ventral BOLD signals was highly robust (*p*<0.001); however, the correlation values of around 0.1 were modest. We argue that this is not a major concern for the following reasons. There were many aspects contributing to the variance of the FFA and PPA BOLD signals difficult to account for, including lack of specificity of the BOLD signal due to temporal smearing and non-neural factors such as respiration or heartbeat. Further, the BOLD signals in FFA and PPA are not driven only by early visual regions: local recurrent activity and higher order regions contribute as well. There were also many aspects contributing to the variance of the occipital alpha signals, including from the noisy MRI scanning environment as well as volume conduction from other nearby sources. Previous studies correlating alpha power in one region to alpha power in another region demonstrated correlation values ranging from 0.1 to 0.2 [48], which also were statistically robust. As this was a correlation of interregional alpha power, it is expected that a correlation of alpha power with the BOLD response would be lower. As such, our findings are comparable to previous reports. Given that our study is correlational in nature, we cannot per se prove that the modulations in the alpha band have a causal consequence for the BOLD signal in the ventral regions. However, several studies using transcranial magnetic stimulation and visual entrainment of 10-Hz activity have demonstrated that the alpha rhythm plays a causal role in the allocation of attention, perception, and working memory maintenance [16],[49]–[51].

Our findings demonstrating that the dorsal attention network is inversely related to the occipital alpha power may be due not only to the control and shifting of spatial attention but also to the one-back working memory component of the task (e.g., [52]), which was object-based in the present study and not spatially based. Furthermore, the occipital alpha activity itself may reflect an aspect of target evaluation and encoding, although this is unknown, as most working memory studies have examined alpha during the delay period and not during processing (e.g., [53]). As our focus was on the gating role of alpha during stimulus processing, we did not probe the working memory delay period per se.

The alpha lateralization was strongest 0.1–1.9 s after stimulus onset, indicating that the alpha modulation was persistent during stimulus presentation (0–2 s) and thus may play an active role in filtering throughout stimulation. While many findings of alpha lateralization effects on behavior involved anticipatory alpha activity (i.e., in a cue-target interval) [7],[8],[30], we demonstrated that alpha activity not only served to initially gate a stimulus, but also operated as a persistent gating mechanism during sustained attention and stimulation. This persistent gating is indeed relevant for real-life situations, in which visual input is always present and endogenous attention operates continuously. The alpha lateralization allowed two spatially separable signals from the EEG data to be correlated with the BOLD data. The finding of a negative correlation between alpha power and the BOLD signal (Figure S3) is well established [25]–[28]; however, we extend previous findings to show a spatial distinction of the alpha power in nearby, but functionally separate, regions and its negative correlation with the BOLD signal (Figure 4).

We speculate that alpha, under top-down control, first serves to gate sensory information in early visual regions. This has then subsequent effects on the neural activity in downstream object-selective regions. The notion of directionality is, however, difficult to substantiate from the data since the transmission delay between early visual and ventral regions might be on the order of 40–60 ms [54]. Due to the hemodynamic response function, the BOLD signal peaks 4–5 s after neuronal activation, whereas the changes in alpha band activity are instantaneous. Since we do not know the hemodynamic response function with great accuracy in extrastriate cortex, it is problematic to determine whether the modulation in the alpha activity actually preceded the BOLD modulation. Future work applying intracranial recordings in non-human primates or electrocorticography recordings in humans would be required to test the question of directionality. Our results also extend previous findings on object- and feature-based attention [44],[55],[56]. We now have shown that a spatial cue, in a scene in which object type is uncorrelated with position in the visual field, can modulate activity in object-selective areas. Furthermore, not only was activity representing the category of the attended object boosted, but also the activity representing the category of the distracting object was reduced. Also, in distinction from other studies (e.g., [57]), the modulation of activity in the feature/object-selective region that we observed was in a cortical region separate from where the modulation of alpha power with attention was observed. Interestingly, Ester et al. [58] found that features of the item held in working memory spread to retinotopic locations outside of where the stimulus was initially presented. Our findings cannot be directly compared with this result, since in their study the object was an oriented grating (which is represented in early visual regions) and no distractor stimulus was presented in the opposite hemifield. We believe that the ipsilateral alpha power reflecting suppression of the unattended stimulus in our experiment operates in a spatial sense, independent of stimulus target characteristics.

These results extend the functional role of oscillatory brain activity by demonstrating that regionally specific alpha activity is involved in routing of information. In future work it would be of great interest to investigate whether the alpha activity is involved in routing of information in areas other than the visual system. EEG is likely to detect only the strongest neuronal signals, and it remains a possibility that the alpha band activity operates in the full dorsal stream. Further, it is a theoretical possibility that the alpha band activity operates in the ventral stream to selectively modulate processing in FFA and PPA. These possibilities are best investigated using intracranial recordings in either humans or non-human primates. Certainly, in somatosensory regions robust alpha activity is also observed, which is predictive of tactile allocation of attention and performance [59]–[62]. In the auditory system, modulations of alpha activity by attention tasks have been reported as well [63],[64]. In the light of our current findings, this set of studies suggests that alpha activity in sensory regions might play a general role for routing when attention is manipulated. The functional inhibition by the alpha activity might generalize well beyond sensory regions. In a recent monkey study, it was demonstrated that the selection of a given stimulus–response rule was associated with neuron-specific gamma synchronization in prefrontal cortex [65]. Importantly, the deselection of the rule was associated with synchronization in the alpha band in the same neuronal population.

Evidently, the alpha activity we observed in visual regions is under strong top-down control. The mechanisms and regions exercising the top-down control need to be identified [66]. Our finding demonstrating that the dorsal attention network (the right FEF and bilateral IPS) correlated with the occipital alpha power lends support to the notion that the dorsal attention network exercises control over sensory areas [31]. Interestingly, we found that this correlation of the dorsal attention network with occipital alpha power was negative, was stronger with specifically the ipsilateral occipital alpha power, and included a right-lateralized FEF contribution. We suggest that the negative correlation is due to the dorsal attention network exercising control by physiological mechanisms serving to actively decrease alpha activity. The decrease in alpha power then serves to open the information flow by disinhibition. Because the occipital alpha power also correlated with the hemisphere-specific occipital BOLD signal, we cannot rule out that the dorsal attention network first modulated some other aspect of occipital neural activity, which then subsequently affected both the alpha power and the BOLD signal. A recent study [56] found increased gamma band synchronization between the inferior frontal junction and the relevant ventral stream regions in an object-based attention task; routing via early visual regions would not be expected in this higher level feature-based task. However, it may be that the mechanism of control is directly executed by increased long-range synchronization in the alpha band between the dorsal attention network and early sensory regions (e.g., [67],[68]). This is also consistent with recent findings [69] that frontocentral alpha power reflected maintenance of relevant visuospatial locations. Further, the communication between early visual regions and the ventral stream areas might be established by alpha band synchronization. Furthermore, the top-down drive might be exercised directly via neocortex or via subcortical areas. For instance, intracranial monkey recordings have shown that the pulvinar nucleus of the thalamus is coherent with visual regions in the alpha band [18],[70]. The negative correlation between the dorsal attention network and alpha power has also been reported in resting state data studies combining fMRI and EEG [26],[32],[33]. We here extend those findings by demonstrating that a negative correlation also was present in a spatial attention task. This suggests that the dorsal attention network serves to control the visual alpha activity in a top-down manner. One might ask why the alpha power ipsilateral to the direction of attention was more strongly anticorrelated with the dorsal attention network than the alpha power in contralateral regions. We suggest that the reduced alpha power in contralateral regions decreased the signal-to-noise ratio, making it difficult to reliably detect the anticorrelation. Indeed, examining the results of contralateral alpha activity at a more liberal threshold depicted the same pattern of negative correlation with the dorsal attention network, albeit not strong enough to be significant. Finally, our finding of a right-lateralized FEF involvement is supported by previous findings [71]–[73]. In short, our findings suggest that the dorsal attention network controls the engagement of visual regions by actively suppressing the occipital alpha activity; the precise route and mechanism of this control remains to be tested.

In conclusion, the functional role of the alpha band activity has in the past been somewhat underappreciated from a physiological perspective. Our results provide support for the notion that alpha band activity operates in a regionally specific manner, can serve to gate the information flow, and is related to dorsal regions previously identified in top-down control of attention.

## Materials and Methods

### Participants

Sixteen healthy participants (15 females; mean age 21.6 y) participated in the main simultaneous EEG–fMRI study. These were selected from a group of 34 participants (29 female) in which a similar experiment was run collecting EEG data alone, to ensure adequate performance on the task, minimal eye movements during trials, and sufficient alpha lateralization with attention shifts. All participants gave written informed consent, and the study was approved by the national competent ethical reviewing board per the Medical Research Involving Human Subjects Act, Commissie Mensgebonden Onderzoek Regio Nijmegen/Arnhem, registered under dossier number 2001/095.

### Task Design

In both the preliminary EEG-alone experiment and the main EEG–fMRI experiment, we used a one-back working memory task in which only the image on the attended side was to be remembered and compared with the image on the attended side in the next trial. A gray background was present throughout. Each trial (12 s total duration) began with a dark gray “xxx” in the center of the screen, changing to a black “xxx” to indicate to participants not to blink starting at this time (Figure 1C). After 2 s, the black “xxx” changed to either “<x<” or “>x>” with 50% probability and unrelated to the previous trial. The participant must fixate at the center “x” but attend to the side indicated. After 0.5 s, two images appeared at each side of the fixation cross. The cue-target interval from the spatial cue to image onset was 0.5 s, as a minimal amount of time for allocation of spatial attention and alpha lateralization accrual to occur by the time of stimulus onset. However, because of this short interval, we did not expect to see alpha lateralization build up significantly prior to the target stimulus onset.

In all trials, there was one face and one landscape image, with a 50% probability that the attended side was a face (or landscape). Participants were instructed to encode the image on the cued side, compare it to the previously attended image, and remember it as a sample for the next trial (Figure 1B). If the cued picture matched the previously attended picture, the participant should press a button with their right index finger, otherwise do nothing. The central arrows remained while the images were present (total 2 s). Afterwards, a black “xxx” returned, encouraging participants not to blink for 1 s (Figure 1C). It then changed to a dark gray “xxx” for 6.5 s and participants were allowed to blink during this interval.

Of 200 trials total, 160 were not a match, 28 were a one-back match (response required), and the remaining 12 were catch trials. Eight catch trials comprised the non-attended image from the previous trial becoming the attended image on the current trial (no response required), and four catch trials comprised the attended image on the previous trial becoming the non-attended image on the current trial (no response required). The task was divided into four sets of 10 min each, with a 1-min break between sets. The break was primarily to give the participant a temporary pause from the task, but was also used to calibrate the eye position in the eye tracking data. The only differences between the EEG-alone and main EEG–fMRI tasks were as follows. In the preliminary EEG experiment, each 1-min break comprised 12 s of “zxz” (indicating that the participants could close or move their eyes), 12 s of moving “x” around the screen (to aid in eye tracker calibration), 12 s of “zxz”, 12 s of moving “x”, and 12 s of “zxz”. In the main simultaneous EEG–fMRI experiment, each 1-min break comprised 12 s of moving “x”, followed by 48 s of “zxz”. A different set of faces and landscapes was used for the EEG-alone and simultaneous EEG–fMRI experiments.

### EEG–fMRI Acquisition

MRI data were acquired on a 3 T Trio scanner (Siemens) with a 32-channel head coil. After two localizer scans were acquired in order to position slices, the main functional MRI scan was acquired (five-echo echo planar imaging [EPI] with echo times of 6.9, 16.2, 25, 35, and 44 ms; flip angle 80 degrees; 64×64 in-plane voxels; 39 slices; 3.5×3.5×3.0–mm voxel resolution; acceleration factor = 3 with generalized autocalibrating partially parallel acquisition, also known as GRAPPA). The fMRI acquisition sequence was modified to allow a delay (silent period) after a certain number of volumes. Specifically, the repetition time was 2.0 s; however, after every four radiofrequency (RF) excitations per slice, two were skipped (i.e., 4.0-s gap of no RF or gradient changes), during which the face/landscape part of the trial was presented so as to obtain EEG data free from RF or gradient-switching artifacts. Thus, four EPI volumes were collected per trial, beginning at 2.5, 4.5, 6.5, and 8.5 s after the onset of the stimulus images (Figure 1B and 1C). The two EPI volumes that would have occurred at 0.5 s and 10.5 s after image onset were the two that were skipped.

A separate functional localizer task (5 min, 36 s) consisting of a rapidly presented series of centrally presented faces or landscapes in a block design, identical to that used in a previous study [74], was run in the same MRI session to obtain participant-specific FFA and PPA regions. This separate functional localizer used the same EPI parameters as the main task but without the MRI silent period. The faces and landscapes in the functional localizer task were also different from both the main task in the EEG–fMRI experiment and the preliminary EEG-alone task. Finally, a structural MRI was acquired of each participant (3-D gradient-recalled inversion recovery, inversion time = 1,100 ms, repetition time = 2,300 ms, echo time = 3.03 ms, flip angle = 8 degrees, 192 slices, 256×256 per slice, 1×1×1–mm resolution).

An MRI-compatible EEG cap with 63 EEG channels plus one electrocardiogram channel was used with a 5,000-Hz sampling rate, with hardware filter settings of 250-Hz low-pass and a high-pass 10-s time constant (BrainCap; Brain Products, Easycap; extended international 10–20 layout). The MRI-compatible amplifiers were placed out of the MRI, near the participant's body. A Polhemus FASTRAK device was used to record the exact location of each EEG electrode on the participant's head relative to three fiducial points. An infrared eye tracker (50-Hz sampling rate) was used to monitor eye movements. Files of raw data plus explanatory files for the EEG–fMRI experiment are available from the Dryad database (http://dx.doi.org/10.5061/dryad.2hr38).

### fMRI Preprocessing

The multi-echoes of both the main fMRI and localizer task were combined using a linear weighting of the echoes based on signal-to-noise ratio [75]. This procedure simultaneously applied rigid-body motion correction. Using SPM8 (http://www.fil.ion.ucl.ac.uk/spm), spatial smoothing was applied to the localizer EPI volumes with a full-width at half-maximum of twice the voxel size. The eighth EPI volume from the functional localizer task was spatially aligned to the eighth EPI volume of the main task in order to match spin history effects (considering the 4-s gap in the main task); the same transformation was applied to all the localizer EPI volumes. Next, the fifth EPI volume of the main task (with greater T1 weighting) was then spatially aligned to the structural MRI of that participant; the same transformation was applied to all EPI volumes. The structural MRI was then non-rigidly warped to the MNI T1 template; the same warping was applied to all functional EPI volumes.

### fMRI Ventral Region Analysis

To obtain the ventral functional ROIs, statistical *t*-tests contrasting the face blocks with the landscape blocks in the localizer task were computed in a GLM with fixation-only blocks as well as motion parameters included as regressors of no interest [74]. Since the voxels found significant at a threshold of *p*<0.05 (FWE corrected) in some cases resided outside of inferior temporal cortex (e.g., parietal or frontal cortex), the significant voxel mask was further masked by regions of “FFA” and “PPA” obtained from the Neurosynth database (http://neurosynth.org/). We excluded the dorsal object-selective regions in order to exclude viewpoint-specific activations [76]. From this dual mask of voxels, the time courses from the PPA and FFA were extracted (Figure S3). The BOLD response per trial was computed from the average of the EPI volumes beginning 4.5 s and 6.5 s after face/landscape stimulus onset. These time points were determined as those for which the greatest modulation of attention between faces and landscapes occurred (Figure S3C and S3D).

Activity per trial was averaged over all voxels within a region for a given participant. The BMI was computed per participant (Figure 3) to demonstrate the effects of attention on the FFA and PPA. The values of BMI over participants for a given region were tested for a difference from zero in a two-sided *t*-test (*p*<0.05).

### EEG Analysis

Preprocessing and source localization of EEG data was computed using the FieldTrip toolbox [77] (http://fieldtrip.fcdonders.nl). Trials and channels with excessive artifacts were rejected; subsequently, the data was rereferenced to the average of all remaining channels and down-sampled to 500 Hz. A three-shell boundary element model precomputed based on a template segmented T1 brain (segmentation from BrainWeb; http://brainweb.bic.mni.mcgill.ca/brainweb/anatomic_normal.html) was used as the volume conductor model [78]. Rather than using template electrode positions, the electrode positions recorded per participant were first coregistered to the individual participant's MRI; subsequently, they were transformed to MNI coordinates via the linear transformation obtained from coregistration of the participant-specific MRI to MNI space. The forward lead field was then computed with a grid resolution of 8 mm. Subject-specific individual alpha frequencies showing the greatest modulation of alpha power with attention condition were determined based on preliminary channel-level analysis of the time–frequency representation. This preliminary analysis included the application of a denoising source separation method [79] (http://www.cis.hut.fi/projects/dss) for removing ballistocardiogram artifact components. The peak alpha frequency was found at 9 Hz in 3/16 participants, 10 Hz in 11/16 participants, and 11 Hz in 2/16 participants.

The alpha power was then projected to source space using a frequency domain beamformer [80] (Figure 2A). Channel-level EEG can be considered a mixture of many different brain and artifact sources. The key feature of the beamformer approach is to operate as a spatial filter that enhances the signal from a given region, here early visual cortex, while suppressing activity from other sources including artifacts. While the channel-level analysis did reveal significant hemispheric alpha lateralization, the signal-to-noise ratio was improved by applying the beamformer source analysis [81]. Indeed, the beamformer successfully suppressed the ballistocardiogram artifact without additional processing. The voxels in posterior cortex (defined as MNI *y*-coordinate less than zero) exhibiting a maximal (left) and minimal (right) AMI value were used for further analysis. First, the alpha power per trial was extracted for correlation with the fMRI data. Second, the time–frequency representations of the task effects at these source locations were computed with a frequency-specific beamformer (Figure 2B and 2C) to show frequency and temporal specificity.

### Correlation between Occipital EEG and Occipital fMRI

The trial-by-trial Pearson correlation of alpha power and BOLD signal within a participant was computed. Four different correlations were computed: separately from each hemisphere in visual cortex and separately for each spatial attention condition. The alpha power was extracted from the voxel in each participant of maximal AMI in the left hemisphere and minimal AMI in the right hemisphere. The BOLD signal was extracted from voxels within a 1-cm radius of this location, defined by the EEG and averaged over voxels. The BOLD response per trial was computed from the average of the EPI volumes beginning 4.5 s and 6.5 s after picture onset. For a given correlation, all valid trials were used (valid trials were defined as those with a correct response of non-match and not rejected for artifacts). The values of correlation over all participants were tested in a two-sided *t*-test (*p*<0.05 threshold, Bonferroni corrected).

### Correlation between Occipital EEG and fMRI in Object-Selective Ventral Regions

The trial-by-trial Spearman correlation of alpha power and BOLD within a participant was computed. For a given correlation, all valid trials were used (valid trials were defined as those with a correct response of non-match and not rejected for artifacts). On each trial, the alpha power was calculated from only either the contralateral or ipsilateral hemisphere, and the BOLD amplitude was derived only from the region of the attended or unattended object category (from the functional localizers). The values of correlation over all participants were tested in a two-sided *t*-test (*p*<0.05 threshold, Bonferroni corrected). To assess whether the regions were mutually correlated, the four values (contralateral alpha power, ipsilateral alpha power, BOLD_attended_, and BOLD_unattended_) were further compared using a partial Spearman correlation. For a given pair (e.g., alpha_ipsi_ and BOLD_unattended_) to correlate, the other two values (alpha_contra_ and BOLD_attended_) were included to be controlled for.

### Assessment of Networks Correlating with Occipital Alpha

To query which putative control regions related to the occipital alpha activity, irrespective of attended hemifield or object category of attention, we fitted a GLM to the data using SPM8, which included all task regressors, regressors explaining non-task variance, and regressors explaining alpha power modulations. We used the GLM framework in the SPM8 software to take advantage of the built-in routines for correction for multiple comparisons in a whole-brain analysis. Task regressors and alpha-power-based regressors were created using the canonical hemodynamic response function in SPM8. We accounted for the two missing EPI volumes at the moment the trial was presented by deleting the values in the regressors after convolution with the hemodynamic response function. The seven task regressors included trials of (1) attend right face, (2) attend left face, (3) attend right landscape, (4) attend left landscape, (5) one-back matches, (6) the first type of catch trial, and (7) the second type of catch trial. The non-task variance regressors included six motion regressors, a white-matter seed time course, a cerebrospinal fluid seed time course, and four regressors modeling each of the positions of the scans relative to the 4-s scan gap between each trial to account for T1 effects. Finally, the four alpha regressors contained the estimated power from the spatial peak in each occipital hemisphere separately and for each direction of spatial attention separately, i.e., (1) from the left during attend-left trials, (2) from the right during attend-right trials, (3) from the left during attend-right trials, and (4) from the right during attend-left trials. The four alpha regressors were created by dividing the alpha power by the mean over all trials, and were included as a parametric modulator of attend-left and attend-right trials so that the hemodynamic response was convolved with the alpha power per trial. However, these two conditions themselves (attend left and attend right) were not included in the model as regressors because the variance explained by them can be fully accounted for by regressors 1–4.

To confirm that the GLM (after mock GLM and deletion of rows) was set up correctly and that regressors for non-task variance were appropriately included, a *t*-test was performed to contrast attention to the left (attend left face and attend left landscape) versus attention to the right (attend right face and attend right landscape); results are shown in Figure S5. The main contrasts of interest included the alpha power regressors, namely, all four alpha power regressors together, only the two ipsilateral regressors, and only the two contralateral regressors. All results were thresholded by first setting a voxel-uncorrected threshold of *p*<0.005 followed by a cluster-level size threshold to correspond to *p*<0.05 (FWE corrected).

## Supporting Information

Figure S1 **Effect of spatial attention on BOLD activity in visual regions.** Each bar represents the average over participants (with standard error) of the difference between conditions of attend left minus attend right, in percent change from baseline (asterisk indicates significantly different from zero, *p*<0.05, Bonferroni corrected for testing 14 regions). LCal, left calcarine; LCun, left cuneus; LFus, left fusiform; LIOcc, left inferior occipital; LLing, left lingual; LMOcc, left middle occipital; LSOcc, left superior occipital; RCal, right calcarine; RCun, right cuneus; RIOcc, right inferior occipital; RFus, right fusiform; RLing, right lingual; RMOcc, right middle occipital; RSOcc, right superior occipital.
(PDF)

Figure S2 **Regions of interest from FFA and PPA per participant.** Red shows the FFA ROI and blue shows the PPA ROI. Each ROI is determined by first using the localizer task in the contrast of faces with landscapes, followed by additional masking with an anatomical ROI from the Neurosynth database with search terms “FFA” and “PPA”.
(PDF)

Figure S3 **Time courses of ventral BOLD signals.** (A) BOLD signal from FFA (percent change from pre-task baseline, offset removed relative to the first time point at 3.5 s) for attended faces (solid) and attended landscapes (dashed). (B) BOLD signal from PPA (percent change from pre-task baseline, offset removed relative to the first time point at 3.5 s) for attended faces (solid) and attended landscapes (dashed). (C) BOLD signal from FFA, as the difference in the percent change from pre-task baseline between attended faces and landscapes within a participant, averaged over participants. (D) BOLD signal from PPA, as the difference in the percent change from pre-task baseline between attended faces and attended landscapes within a participant, averaged over participants. For all panels, the time points against which the data are plotted refer to the middle of the time when a given EPI volume was acquired (e.g., the volume beginning at 2.5 s and ending at 4.5 s is plotted at 3.5 s). Error bars indicate standard error of the mean over participants.
(EPS)

Figure S4 **Correlation of alpha power and BOLD signal from the same region in visual cortex.** The trial-by-trial correlation for each spatial attend condition was considered separately, and the activity in each hemisphere was also considered separately. The location was chosen based on the peak coordinate in each participant of maximal AMI for the left hemisphere and minimal AMI for the right. Each bar is the mean over participants (error bar is standard error). All correlations are significantly negative (*p<0.005) except that in the left occipital region in the left attention condition. LOcc, left occipital; ROcc, right occipital.
(EPS)

Figure S5 **Regions showing significant modulation with the task of shifting attention to the left or right.** Assessed in the GLM framework using SPM8 (*p*<0.005 uncorrected initial voxel threshold, followed by *p*<0.05 FWE cluster-level threshold). These results act as a control analysis for the analysis including the alpha power as regressors in the GLM framework. (A) BOLD activity for attention to the left contrasted with attention to the right. (B) BOLD activity for attention to the right contrasted with attention to the left.
(EPS)

## Abbreviations

AMI: alpha modulation index
BMI: blood-oxygenation-level-dependent modulation index
BOLD: blood-oxygenation-level-dependent
EEG: electroencephalography
EPI: echo planar imaging
FEF: frontal eye field
FFA: fusiform face area
fMRI: functional magnetic resonance imaging
FWE: family-wise error
GLM: general linear model
IPS: intraparietal sulcus
MNI: Montreal Neurological Institute
PPA: parahippocampal place area
RF: radiofrequency
ROI: region of interest
