# Supplementary figures and images for: Occipital Alpha Activity during Stimulus Processing Gates the Information Flow to Object-Selective Cortex
Source: PLoS Biol. 2014 Oct 21;12(10):e1001965. doi: 10.1371/journal.pbio.1001965 (PMC4205112; doi:10.1371/journal.pbio.1001965)

BOLD Contrast per AAL region averaged over subjects

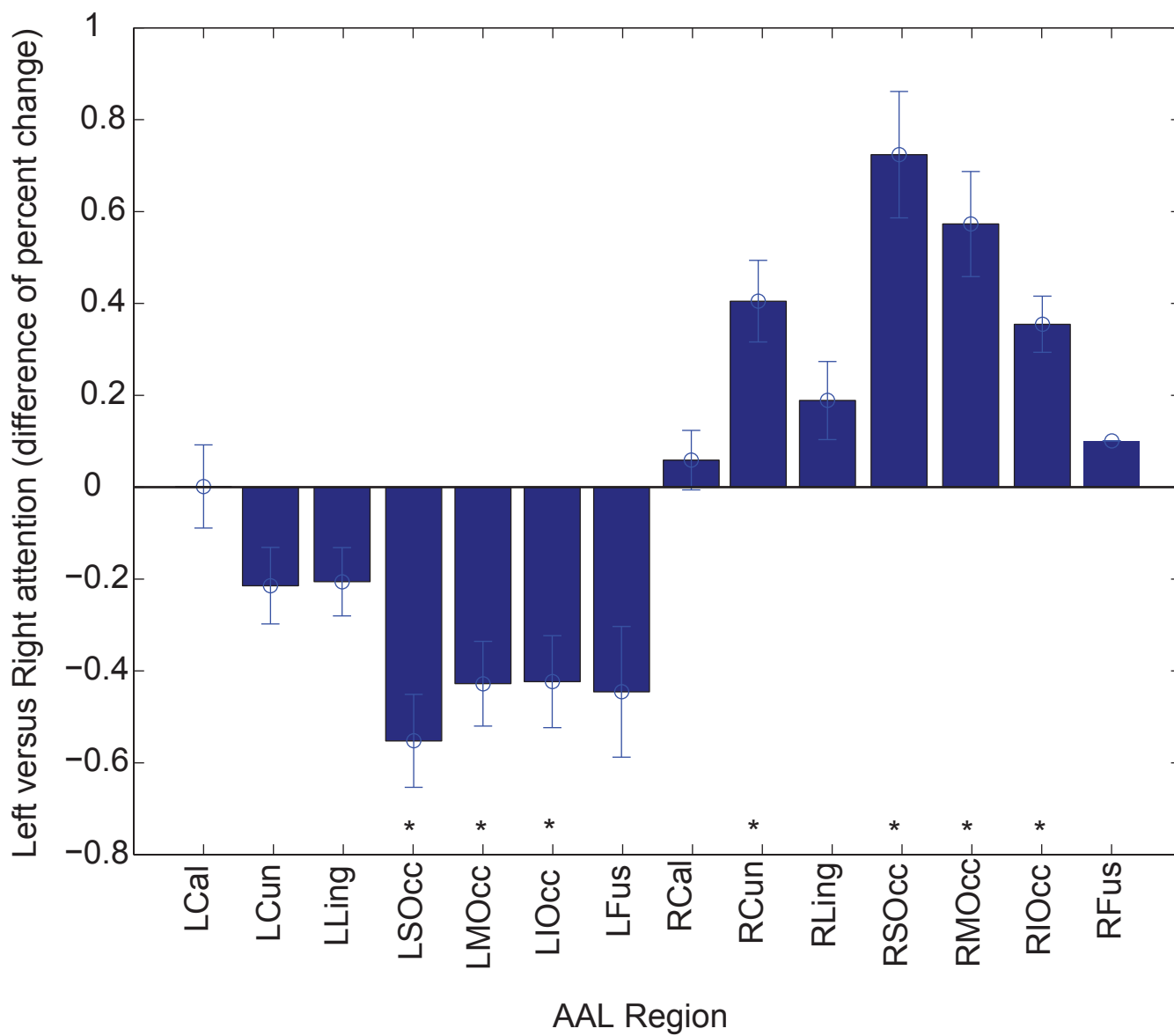

Supplement: Figure S1 — Effect of spatial attention on BOLD activity in visual regions. Each bar represents the average over participants (with standard error) of the difference between conditions of attend left minus attend right, in percent change from baseline (asterisk indicates significantly different from zero, p<0.05, Bonferroni corrected for testing 14 regions). LCal, left calcarine; LCun, left cuneus; LFus, left fusiform; LIOcc, left inferior occipital; LLing, left lingual; LMOcc, left middle occipital; LSOcc, left superior occipital; RCal, right calcarine; RCun, right cuneus; RIOcc, right inferior occipital; RFus, right fusiform; RLing, right lingual; RMOcc, right middle occipital; RSOcc, right superior occipital. (PDF) [file pbio.1001965.s001.pdf]

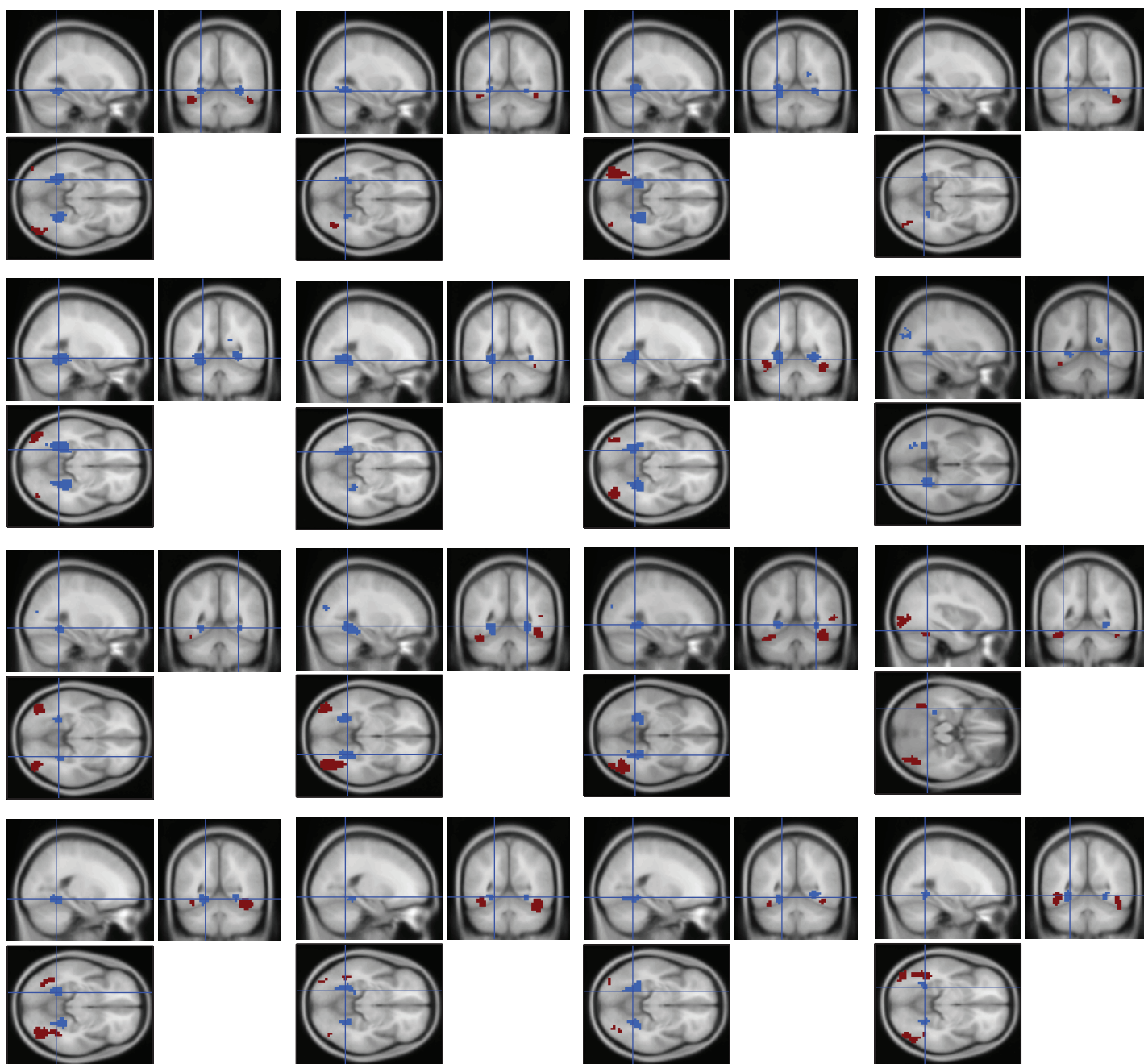

Supplement: Figure S2 — Regions of interest from FFA and PPA per participant. Red shows the FFA ROI and blue shows the PPA ROI. Each ROI is determined by first using the localizer task in the contrast of faces with landscapes, followed by additional masking with an anatomical ROI from the Neurosynth database with search terms “FFA” and “PPA”. (PDF) [file pbio.1001965.s002.pdf]
